# Transient but not permanent benefit of neuronal progenitor cell therapy after traumatic brain injury: potential causes and translational consequences

**DOI:** 10.3389/fncel.2014.00318

**Published:** 2014-10-14

**Authors:** Marco Skardelly, Khaled Gaber, Swen Burdack, Franziska Scheidt, Martin U. Schuhmann, Heidegard Hilbig, Jürgen Meixensberger, Johannes Boltze

**Affiliations:** ^1^Department of Neurosurgery, University of LeipzigLeipzig, Germany; ^2^Institute of Anatomy, University of LeipzigLeipzig, Germany; ^3^Fraunhofer Institute for Cell Therapy and Immunology and Translational Centre for Regenerative Medicine, University of LeipzigLeipzig, Germany; ^4^Stroke and Neurovascular Regulation Laboratory, Massachussets General Hospital and Harvard Medical SchoolCharlestown, MA, USA

**Keywords:** neural progenitor cells, cell therapy, TBI, long-term functional outcome, translational research

## Abstract

**Background**: Numerous studies have reported a beneficial impact of neural progenitor cell transplantation on functional outcome after traumatic brain injury (TBI) during short and medium follow-up periods. However, our knowledge regarding long-term functional effects is fragmentary while a direct comparison between local and systemic transplantation is missing so far.

**Objectives**: This study investigated the long-term (12 week) impact of human fetal neuronal progenitor cell (hNPC) transplantation 24 h after severe TBI in rats.

**Methods**: Cells were either transplanted stereotactically (1 × 10^5^) into the putamen or systemically (5 × 10^5^) via the tail vein. Control animals received intravenous transplantation of vehicle solution.

**Results:** An overall functional benefit was observed after systemic, but not local hNPC transplantation by area under the curve analysis (*p* < 0.01). Surprisingly, this effect vanished during later stages after TBI with all groups exhibiting comparable functional outcomes 84 days after TBI. Investigation of cell-mediated inflammatory processes revealed increasing microglial activation and macrophage presence during these stages, which was statistically significant after systemic cell administration (*p* < 0.05). Intracerebral hNPC transplantation slightly diminished astrogliosis in perilesional areas (*p* < 0.01), but did not translate into a permanent functional benefit. No significant effects on angiogenesis were observed among the groups.

**Conclusion:** Our results suggest the careful long-term assessment of cell therapies for TBI, as well as to identify potential long-term detrimental effects of such therapies before moving on to clinical trials. Moreover, immunosuppressive protocols, though widely used, should be rigorously assessed for their applicability in the respective setup.

## INTRODUCTION

Acute traumatic brain injury (TBI) is followed by a complex cascade of pathophysiological sequelae such as excitotoxicity, formation of free radicals, release of inflammatory molecules, as well as diffuse axonal and neuronal injury. These processes exacerbate blood–brain barrier (BBB) breakdown, activate micro-(Napoli and Neumann, 2009) and astroglia (Silver and Miller, 2004) and enhance leukocyte migration to the lesion site, thus ultimately cumulating into subacute (“secondary”) brain damage (McIntosh et al., 1996; Teasdale and Graham, 1998) with a net loss of cerebral tissue and function.

However, regenerative processes parallel the emerging tissue damage. Glial cells were shown (i) to limit expansion of brain damage by forming a glial scar, (ii) to partly restore the BBB (Stichel and Müller, 1998; Smith, 2003), (iii) to remove cell debris and apoptotic cells (Napoli and Neumann, 2009; Walter and Neumann, 2009), (iv) to control the adaptive immune system by influencing antigen presentation (Mrass and Weninger, 2006), and (v) to exert neuroprotective and -restorative effects (Napoli and Neumann, 2009) such as angiogenesis (Xiong et al., 2010). Interestingly, endogenous neurogenesis (Gage, 2000) may be of limited relevance for recovery. Neuronal precursor proliferation is promoted after TBI, but it is still uncertain whether this results in stable or functionally relevant neurogenesis (Richardson et al., 2010; Gao and Chen, 2013).

Anti- and pro-regenerative phenomena seem to be closely linked on a molecular and cellular level while occurring simultaneously over days and even weeks after the initial insult. This provides a promising opportunity for novel therapeutic approaches aiming to shift these processes in favor of neuroprotective and/or - regenerative mechanisms. Particularly, the paucity of endogenous restorative capacity may be compensated by local transplantation of neural stem and progenitor cells. These might contribute to cell replacement, but tissue restoration by functional engraftment and its impact are discussed controversely (Kernie and Parent, 2010). However, the cells were clearly shown to exert modulatory and supportive (“bystander”) functions such as angio- as well as neurogenesis (Lu et al., 2003; Gao et al., 2006), and to modulate microglial (Mosher et al., 2012) as well as T-cell reactivity (Wang et al., 2009) – a possible key element for functional and structural brain repair (Kokaia et al., 2012). Although partially abolished after TBI, the immune-privileged state of the central nervous system (CNS) in means of both, innate and adaptive immune responses (Carson et al., 2006), is considered a favorable environment for cells transplanted to support endogenous repair processes.

Nevertheless, transplantation experiments addressing the therapeutic potency of a particular stem cell population need to take into account the clinical situation in terms of the applied transplantation paradigm, relevant endpoint measurements as well as the overall examination period. Particularly, the majority of studies investigated the therapeutic benefit in short- (up to 2 weeks) or medium-term (2–6 weeks) observation periods [for overview see (Schouten et al., 2004; Harting et al., 2008)], while our knowledge regarding long-term (6–12 or more weeks) therapeutic impact of cell therapies is limited to rare studies focusing on bone marrow mesenchymal (Mahmood et al., 2005, 2006) and neural progenitor cell (NPC) populations (Skardelly et al., 2011; Riess et al., 2002). This is of particular importance since long-term functional outcome may significantly differ from that observed during shorter surveillance periods as reported for experimental treatment approaches after stroke (Ji et al., 2009) and Parkinson’s disease (Yang et al., 2009).

Consequently, this study was designed to assess the therapeutic potency of human fetal NPCs after TBI for up to 12 weeks, as well as to evaluate the influence of systemic and local NPC transplantation on post-traumatic functional regeneration, glial activity, immigration of inflammatory cells, and vascularization at different time points.

## MATERIALS AND METHODS

### CELL CULTURE

Human fetal NPC (hNPCs; provided by Prof. Dr. Schwarz, Department of Neurology, University Clinic of Leipzig, Germany) were grown and prepared for transplantation. For a pilot hNPC tracking study, cells were stained with the red fluorescent *in vivo* marker TAMRA (20 mM; Sigma–Aldrich) according to the manufacturer’s instructions prior to administration. For the experimental main series, cells were not labeled with a fluorescence dye, but identified with a human-specific antibody.

Cells were grown to adherence in cell culture flasks at 37°C in 5% CO2 and 5% O_2_. Growth medium contained 2% B-27 supplement (without vitamin A; Invitrogen, Darmstadt, Germany), recombinant human basic fibroblastic as well as epidermal growth factor (both 20 ng/mL; PeproTech, London, UK), and gentamicin (10 mg/mL; Bio-chrom AG, Berlin, Germany) in DMEM-F12. Poly-L-ornithine (15 mg/mL; Sigma-Aldrich, Steinheim, Germany) and human plasma fibronectin (4 mg/mL; Millipore, Taunus, Germany) were used for coating cell culture flasks. Every 7 days cells were passaged by incubation with Accutase®; (PAA Laboratories, Pasching, Austria) at 4°C for 10 min and reseeded at a density of 2.5 × 10^4^ cells/cm2. Cells were dissociated with Accutase®; and assessed for viability by the trypan blue exclusion method prior to transplantation. Only samples with > 90% viable cells were used. The desired numbers of viable cells (1 × 10^5^ for local and 5 × 10^5^ for systemic transplantation, respectively) were dissolved in PBS immediately before local and systemic administration. TAMRA staining was performed according to manufacturer’s (Sigma Aldrich) instructions.

### ANIMALS AND EXPERIMENTAL GROUPS

All animal experiments were authorized by the responsible governmental authorities (license number 26/04). Male Sprague-Dawley rats (*n* = 72) weighing 280–320 g were kept under a 12 h-light–dark cycle at constant humidity and temperature with free access to food and water. All animals received an antibiotic prophylaxis with chloramphenicol (75 mg/kg/day, dissolved in drinking water), starting immediately after TBI. Immunosuppressive treatment [subcutaneous cyclosporine A (CsA), 10 mg/kg; Novartis, Basel, Switzerland] was initiated after TBI in all animals and maintained throughout the study.

The pilot cell tracking study was conducted in six animals, which were allocated to either local or systemic transplantation (see below) of TAMRA-stained hNPCs 24 h after TBI (*n* = 3 each). Rats were sacrificed another 24 h later and immunohistochemically examined for graft detection.

**FIGURE 1:**
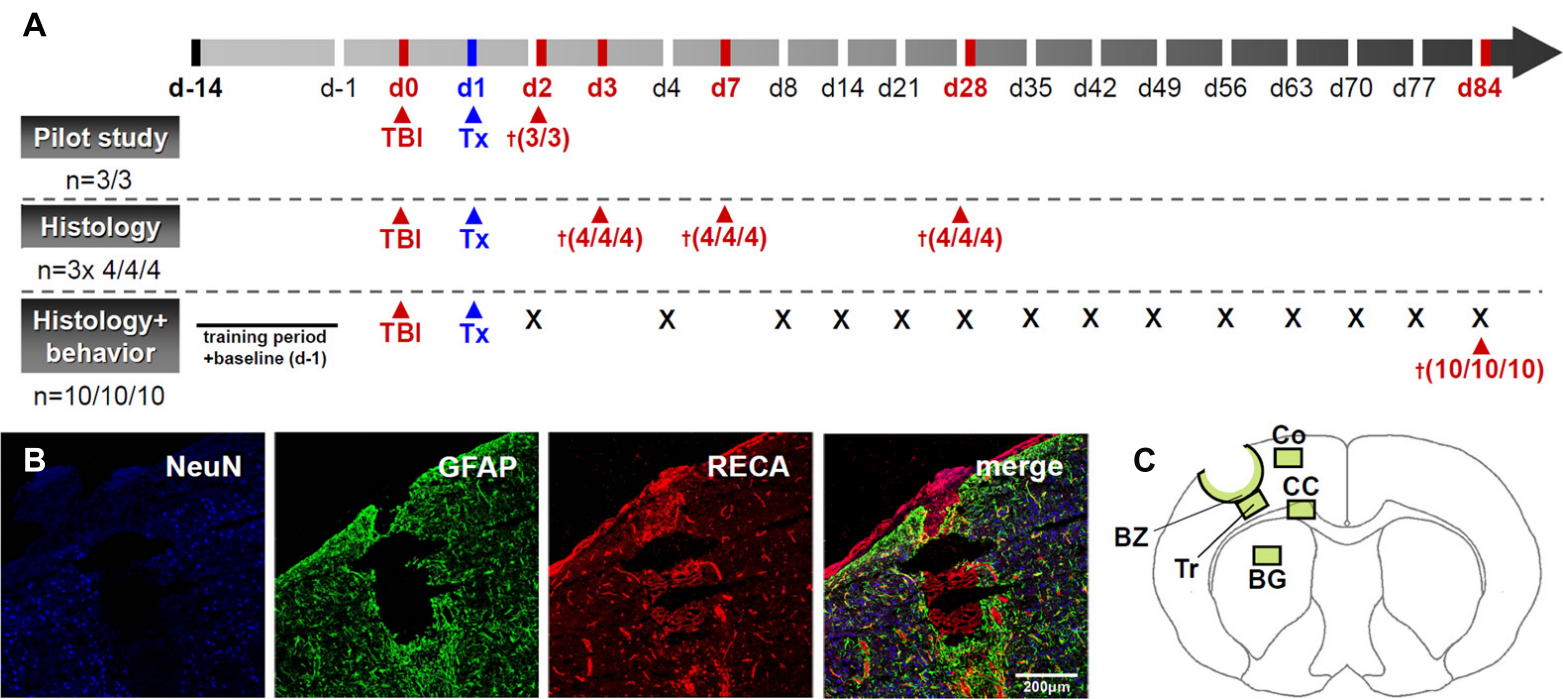
**Outline of the experimental design. (A)** provides a scheme drawing illustrating the experimental plan. “Tx” indicates transplantation time points while the cross sign indicates time points of animal sacrifice. A representative image of the lesion after experimental TBI is given in **(B)** and areas investigated during histological assessments are indicated in **(C)**. BZ, border zone; Tr, trauma; CC, corpus callosum; Co, perilesional cortex; BG, basal ganglia. Scale bar represents 200 μm.

The remaining rats (*n* = 66) were randomly assigned to three experimental groups (*n* = 22 each): controls, systemic (5 × 10^5^ cells) and local transplantation (1 × 10^5^ cells). Each group was randomly split into four subgroups for immunohistochemical investigation at 3, 7, 28 (*n* = 4 each) and 84 days (*n* = 10) after TBI according to **Figure 1**. Weight of all animals was recorded at the day of surgery and mortality rates were recorded for all groups.

### TRAUMATIC BRAIN INJURY AND CELL TRANSPLANTATION

Traumatic brain injury was induced by controlled cortical impact (CCI) and cell administration was performed 24 h after injury. Prior to each surgical intervention rats were anesthetized by an intramuscular injection of fentanyl (0.005 mg/kg; Ratiopharm, Germany), midazolam (2 mg/kg; Ratiopharm, Ulm, Germany) and medetomidine (0.15 mg/kg; Orion Pharma, Hamburg, Germany). Body temperature was kept at 37°C during surgery using a feedback-controlled heating pad equipped with a thermistor rectal probe (FHC Inc., USA). Briefly, the rat’s head was shaved and placed in a stereotaxic frame. The field for surgery was cleaned with Betaseptic®; (Braun, Melsungen, Germany) before exposition of the skull bone by midline skin incision. A round craniotomy (7 mm) was performed 3.5 mm lateral to Bregma on the left side. TBI was induced by CCI using an electromagnetically controlled piston (5 mm; Custom Design and Fabrication, Virginia Commonwealth University, Richmond, VA, USA), which was placed perpendicularly above the exposed, but intact meninges. The device was calibrated to a impact speed of 4 m/s and a compression of 2.5 mm over 150 ms. Following surgery and skin wound suture, anesthesia was antagonized using naloxone (0.12 mg/kg; Ratiopharm), flumazenil (0.2 mg/kg; Hoffmann-La Roche, Basel, Switzerland) and atipamezole (0.75 mg/kg; Orion Pharma). The overall study design and a typical lesion example following TBI are given in **Figures 1A,B**.

Rats assigned to local hNPC transplantation were re-anaesthetized and placed in a stereotactic frame. A deposit containing 1 × 10^5^ cells (passage 8–12, in 3 μL PBS) and located 3 mm beneath the meningeal layer was placed through the center of the contusion using a 26-gage needle connected to a 10 μL Hamilton syringe. After gentle cell injection over 6 min, the needle was slowly withdrawn over further 4 min. For systemic transplantation and control purposes, animals received 500 μL of either cell suspension (5 × 10^5^ hNPCs, passage 8–12) or PBS via the tail vein.

### FUNCTIONAL TESTS

The modified neurological severity score (mNSS) comprises 14 single items that evaluate motor and sensory function, as well as balance and reflexes (Ji et al., 2009). The test is widely to assess therapeutic impact of experimental procedures after brain injury. The rotarod test is a reliable tool to follow up and evaluate motor function after TBI in rodents (Ji et al., 2009). The rats were placed on a computer-controlled accelerating rotating rod (TSE Systems, Bad Homburg, Germany). The time before dropping off the rod (latency) was measured five times for each rat and latency mean values were recorded for each day. All animals were trained on the rotarod for 2 weeks before baseline latencies were measured 1 day prior to TBI.

The rotarod test were conducted in the long-term subgroups (*n* = 10 each) at days -1, 2, 4, 8, 14, 21, 28, 42, 49, 56, 63, 70, 77, and 84 after TBI after appropriate training. The modified Neurological Severity Score (mNSS) was performed at day -1, 2, 8, 14, 28, 42, and 84. All investigators were blinded to group allocation.

### IMMUNOHISTOCHEMICAL STAINING AND ANALYSIS

#### Tissue preparation

Rats allocated to immunohistochemical analysis were euthanized by an intraperitoneal injection of xylazine (50 mg/kg; Bayer AG, Berlin, Germany) and ketamine (200 mg/kg; Merial, Halbergmoos, Germany) at 24 h (cell tracking pilot study), 3, 7, 28, and 84 days following TBI. After thoracotomy, animals were perfused transcardially with 50 mL ice-cold heparinized PBS, followed by 250 mL 4% paraformaldehyde (PFA) in 0.1 M PBS using an automatic perfusion pump (Braun, Melsungen, Germany). The brains were gently removed and stored in 4% PFA in 1 M PBS for 4 h before being equilibrated in 30% sucrose at 4°C for 48 h. A 1 cm coronal block engulfing the entire trauma region was then cut from each brain and placed in embedding medium (OCT, Sakura Finetek, Germany), frozen in liquid nitrogen-cooled isopentane and kept at –80°C. Whole blocks were cut into 40 μm coronal sections using a cryotome. The slices were stored in an antifreeze solution (30% glycerol, 30% ethylene glycol, 40% aqua bidest, 13 mM monosodium phosphate, and 39 mM disodium phosphate) at -20°C until staining.

#### Tissue staining

All histochemical stainings were performed on 40 μm free-floating coronal sections using appropriate primary antibodies according to **Table 1**. The streptavidin-biotin immunoperoxidase staining method was utilized for visualization in all cases.

**Table 1:** Antibodies used in immunohistochemistry.

Antigen	Target	Source	Dilution	Company and clone
CD11b (Ox42)	Microglia	Mouse	1:200	Chemicon, CBL 1512
CD68 (ED1)	Macrophages	Mouse	1.250	Chemicon, MAB1435
GFAP	Astroglia	Mouse	1:400	Sigma, G3893
HuNu	Human Cells	Mouse	1:100	Chemicon, MAB1281
NeuN	Neurons	Mouse	1:200	Chemicon, MAB377
RECA	Vessels	Mouse	1:250	Serotec, MCA970R
Streptavidin		–	1:100	Invitrogen

For detection of the graft after local and systemic transplantation brain slices were stained with HuNu, an antibody being specific for human nuclei, and further screened for TAMRA staining. *In vitro*-preparation (smears) of hNPCs served as a positive control for human nuclei (HuNu) staining.

#### Immunohistochemical analyses

Brain specimens were assessed semi-quantitatively by light microscopy. For post-processing, Olympus’ (New York, NY, USA) ANALYSIS program was used. Five sections with an intersectional distance of 800 μm, and encompassing the center of the brain injury, were evaluated for each animal. Regions of interest (**Figure 1C**) were investigated by counting of positive events in one field of view (FOV) at 40x magnification. Astro- and microglial/macrophage reactivity in the contusion border zone (BZ), traumatic cortical areas directly below the contusion (trauma, Tr), the perilesional cortex (Co), corpus callosum (CC), and basal ganglia (BG, see **Figure 1C** for localization details) were investigated applying a semi-quantitative neuropathological score (**Table 2**).

**Table 2:** Grading of microglial and astroglial reaction assessed by expression of CD11b and CD68.

Grade	CD11b/GFAP	CD68
0	No reaction	0 pos. cells
1	Slight reaction	<10 pos. cells
2	Moderate reaction	10–50 pos. cells
3	Strong reaction	50–200 pos. cells
4	Very strong reaction	>200 pos. cells

**FIGURE 2:**
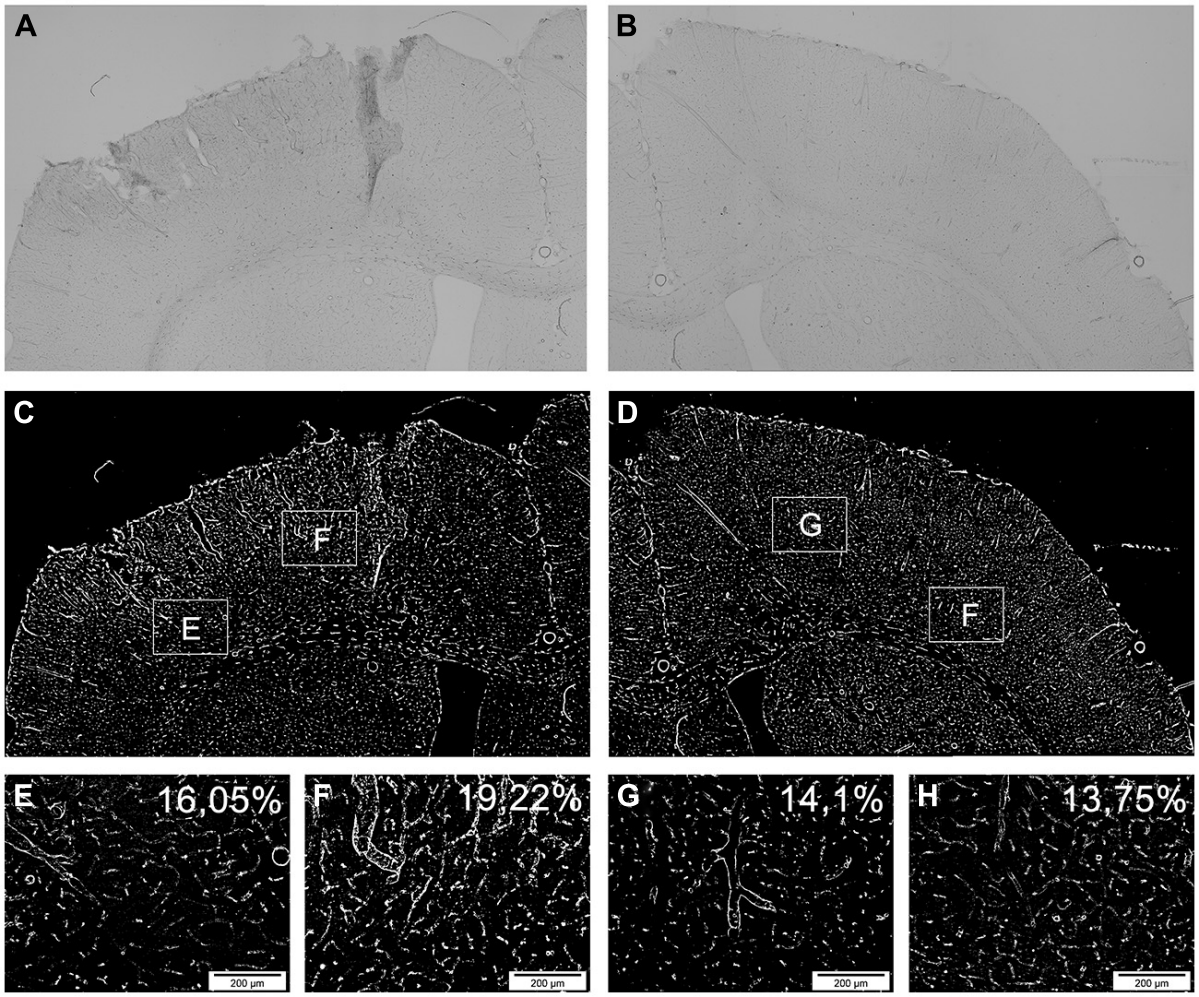
**Determination of vascular changes by RECA.** Demonstration of the vascular reaction by the panvascular marker RECA-1 (rat endothelial cell antigen) 3 days after brain injury. **(A,B)** show an overview of the ipsilateral **(A)** and contralateral **(B)** side of a systemically transplanted animal. Images are acquired by fusion of 9 single images of 2.5x magnification. **(C,D)** demonstrate the same images after binary transformation. **(E–H)** show the lateral **(E)** and medial **(F)** border zone at high power and corresponding contralateral regions **(G,H)**. The RECA-positive proportions are indicated as percentages exhibiting an induction of RECA-expression after brain injury and transplantation **(E–H)**.

Additionally, neuronal (NeuN) and vascular (RECA) compartments were analyzed in the BZ laterally and medially to the lesion as well as in ipsilateral cortical areas. To this end, captured micrographs were converted to binary images (white: stained areas/black: unstained areas). The area of positive structures on the lesioned (ipsilateral) side expressed as percentages and compared between groups (**Figure 2**).

#### Statistical analysis

All data are depicted as mean ± SD. Behavioral data were calculated as percent of d-1 for the rotarod test and derivation to d-1 for the mNSS, respectively. Data obtained at d-1, d2, and d84 were assessed by one-way analysis of variance (ANOVA) or ANOVA on ranks (in case of not normally distributed data) while development of functional impairment over the observation period was investigated by standard area under the curve (AUC) analysis. All histological data were analyzed using one-way ANOVA or ANOVA on ranks tests. The Holm–Sidak method was applied for *post hoc* pairwise multiple comparison procedures; Dunn’s test was used for not normally distributed data. All procedures were performed using the SigmaPlot 11.0 software package (Systat Software, Chicago, IL, USA). Levels of statistical significance are indicated by single (^∗^*p* < 0.05) or double (^∗∗^p < 0.01) asterisks. Due to small sample sizes and thereby limited statistical power, histological data from days 3, 7, and 28 were not subjected to statistical analysis, but only described qualitatively.

## RESULTS

### PILOT STUDY FOR GRAFT IDENTIFICATION

**FIGURE 3:**
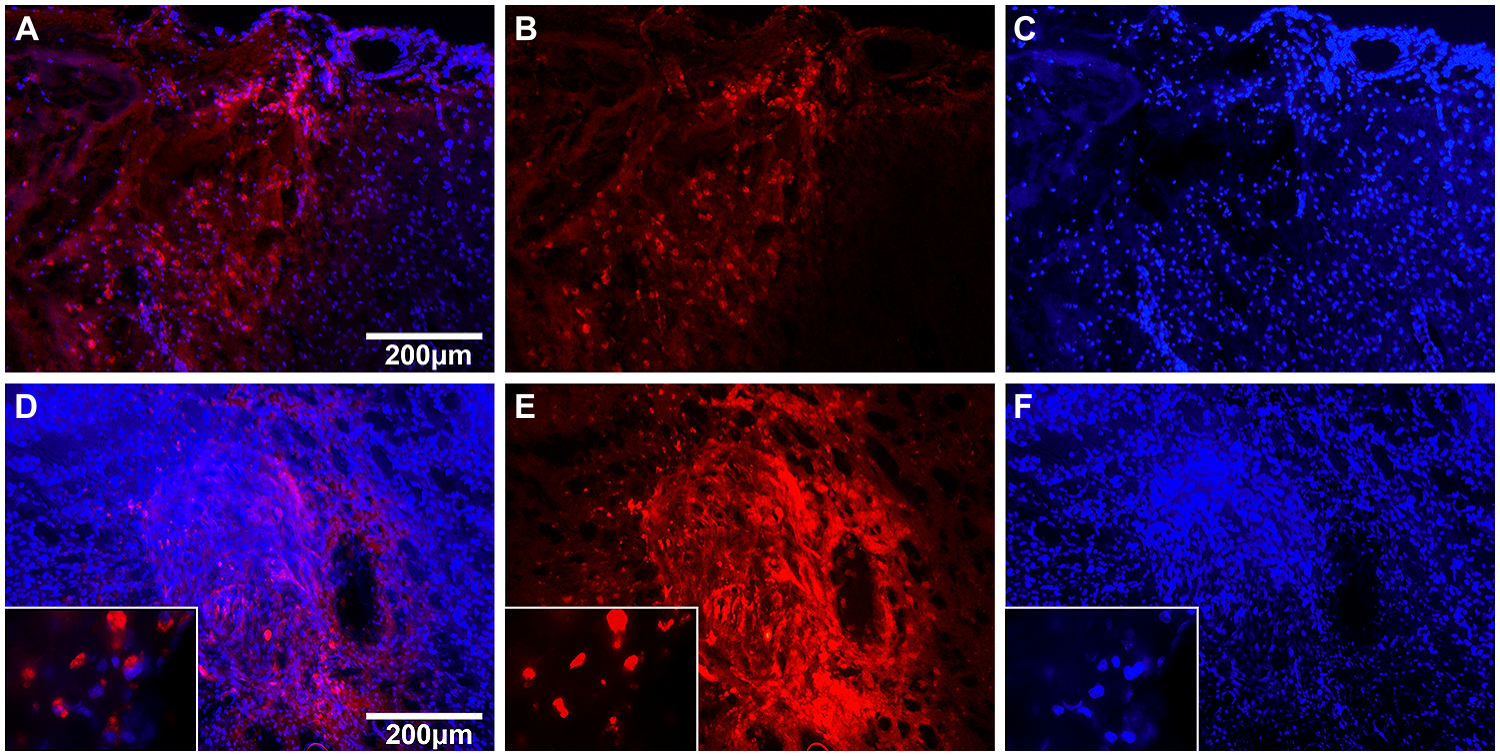
**Cell tracking with TAMRA 24 h after hNPC transplantation.** Fluorescent images of brain slices 24 h after systemic **(A–C)** and local **(D–F)** transplantation of TAMRA-stained human fetal neuronal progenitor cells. Cell nuclei are displayed in blue (DAPI) and TAMRA positive cells in red. **(A–C)**: The left side of images shows the impact area with rarefied cell density. In the center one can identify the border zone with many infiltrated TAMRA-positive cells and on the right side the adjacent unharmed cortex. **(D–F)**: The images display the injection canal with multiple surrounding TAMRA-positive cells. In higher magnification (x100) one can identify single cells and their nuclei.

In a pilot cell detection study, presence of transplanted cell at the lesion site 24 h after local and systemic transplantation was indicated by the TAMRA signal. In animals subjected to local transplantation, numerous TAMRA+ cells were detected in the putamen and in the vicinity of the injection canal (*n* = 3/3). After systemic transplantation, many TAMRA+ cells were identified in the border zone with only a few, scattered cells in the contusion area (*n* = 3/3, see **Figure 3**).

### GENERAL ASPECTS OF TREATMENT SAFETY

We did not observe general signs for safety concerns being related to both treatment procedures, especially no mortality in all groups.

#### Functional outcome

**FIGURE 4:**
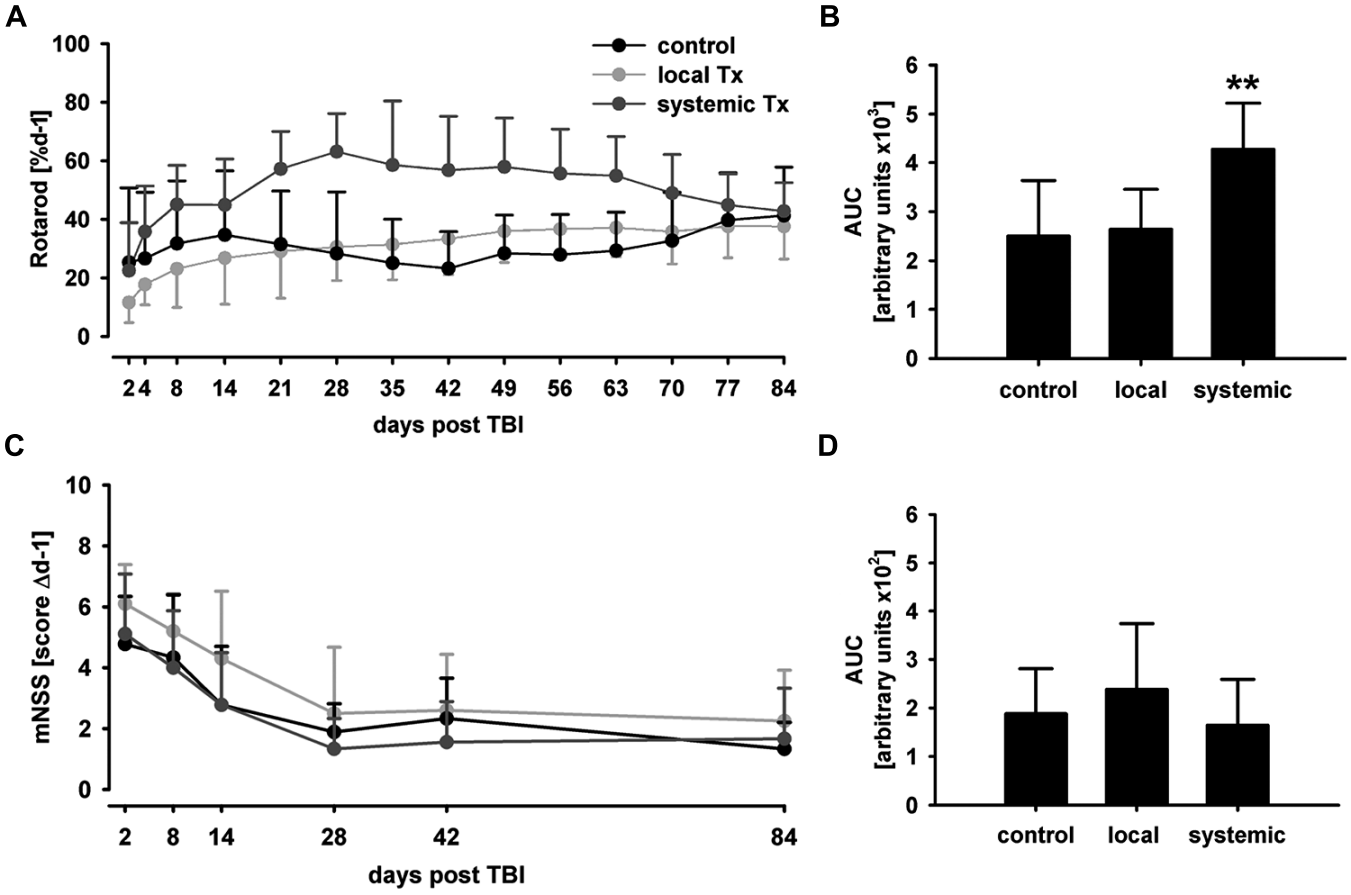
**Behavioral phenotyping after hNPC transplantation.** In the rotarod test, animals subjected to systemic cell transplantation showed a superior performance particularly in the middle of the observation period **(A)**. Despite the functional benefit was transient, it translated into an overall therapeutic effect as indicated by AUC analysis **(B)**. No statistically significant therapeutic effect was observed in the mNSS test although systemically transplanted subjects showed lowest mean AUC values **(C,D)**. **p < 0.01.

There were no statistically significant differences in performance for both behavioral tests among groups in the rotarod and mNSS tests before TBI (d-1, *p* > 0.4, data not shown). Behavioral phenotyping was not possible 24 h after TBI and immediately before hNPC treatment because of significant affection of animals by trauma, which prevented reliable functional assessments. However, no differences were observed between control and systemic transplantation groups at d2 and d4 post TBI in the rotarod (*p* > 0.4 and *p* > 0.1, **Figure 4A**) and at d2 in the mNSS test (*p* > 0.2, **Figure 4C**), arguing against inter-group differences directly after trauma induction.

Expectedly, animals receiving local hNPC transplantation showed lowest mean values in the rotarod and highest scores in the mNSS, but without reaching statistical significance (*p* > 0.05) 1 day after transplantation. This was likely due to the second surgical intervention conducted for local transplantation.

Although slight improvements from baseline values (d2) were observed in locally transplanted animals (*p* > 0.05), rotarod performance did not improve markedly in those animals and controls during the observation period. Systemically transplanted animals showed an overall superior performance as compared to local transplantation and control groups (*p* < 0.01, **Figure 4B**). However, the therapeutic benefit was only transient and all groups showed a comparative rotarod performance between 38 (local transplantation) and 43% (systemic transplantation) of pre-lesion baseline values on d84 (*p* > 0.4).

In accordance to the rotarod test, there was no statistically significant mNSS score difference between the groups at d2 and d84 post transplantation (*p* > 0.1 and *p* > 0.4, **Figure 4C**). Although lowest mean mNSS scores were observed after systemic and highest after local transplantation, there was no statistically significant difference in overall performance among the groups (*p* > 0.1, **Figure 4D**).

#### Histological analysis and cell tracking

In contrast to results of the graft identification pilot study, hNPCs could not be detected after either local or systemic transplantation by the human nuclei antibody (huNu) at any later investigated time-points after d3, although the positive smear control confirmed the functioning of the antibody in every case.

#### Neuronal and vascular compartments

At day 3 after hNPC transplantation, slightly higher NeuN density was observed in locally transplanted subjects. While no obvious differences were observed at days 7 and 28, a reduced density of NeuN-positive cells was observed at day 84 in both, the locally and systemically transplanted subjects (*p* < 0.05 each).

**FIGURE 5:**
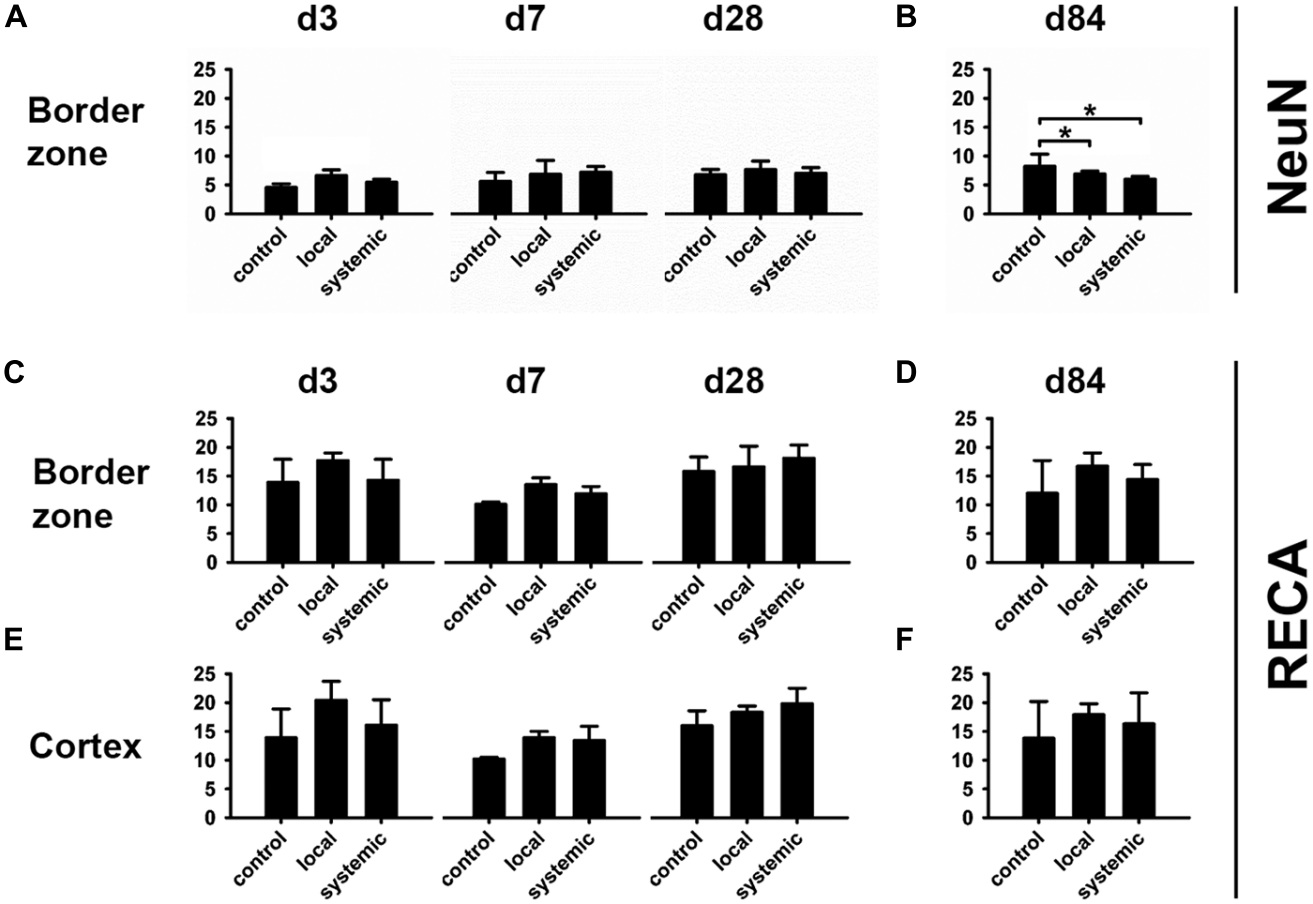
**Changes in the neuronal and vascular compartments after hNPC transplantation.** All information is given in average area percentages of positive staining in investigated micrographs. Higher numbers of NeuN+ cells were observed 3 days after local hNPC transplantation **(A)**, but not thereafter. At day 84, animals receiving systemic or local cell transplantation presented lowest density of NeuN+ cells in the lesion border zone **(B)**. Despite highest mean density of RECA+ cells at day 84 after local transplantation, there was no statistically significant intergroup difference in vascularization **(C–F)**. *p < 0.05.

Transplantation of hNPC slightly increased RECA expression in comparison to control conditions with higher mean percentage of RECA+ structures being observed in both transplantation groups and at any investigated time point in the BZ and cortical areas (**Figures 5C–F**). Most RECA+ vessels were seen in animals subjected to local transplantation. However, statistically significant differences among the groups were not detected at d84 (*p* > 0.05, **Figure 5D**).

#### Impact of cell transplantation on macrophages and microglial activity

**FIGURE 6:**
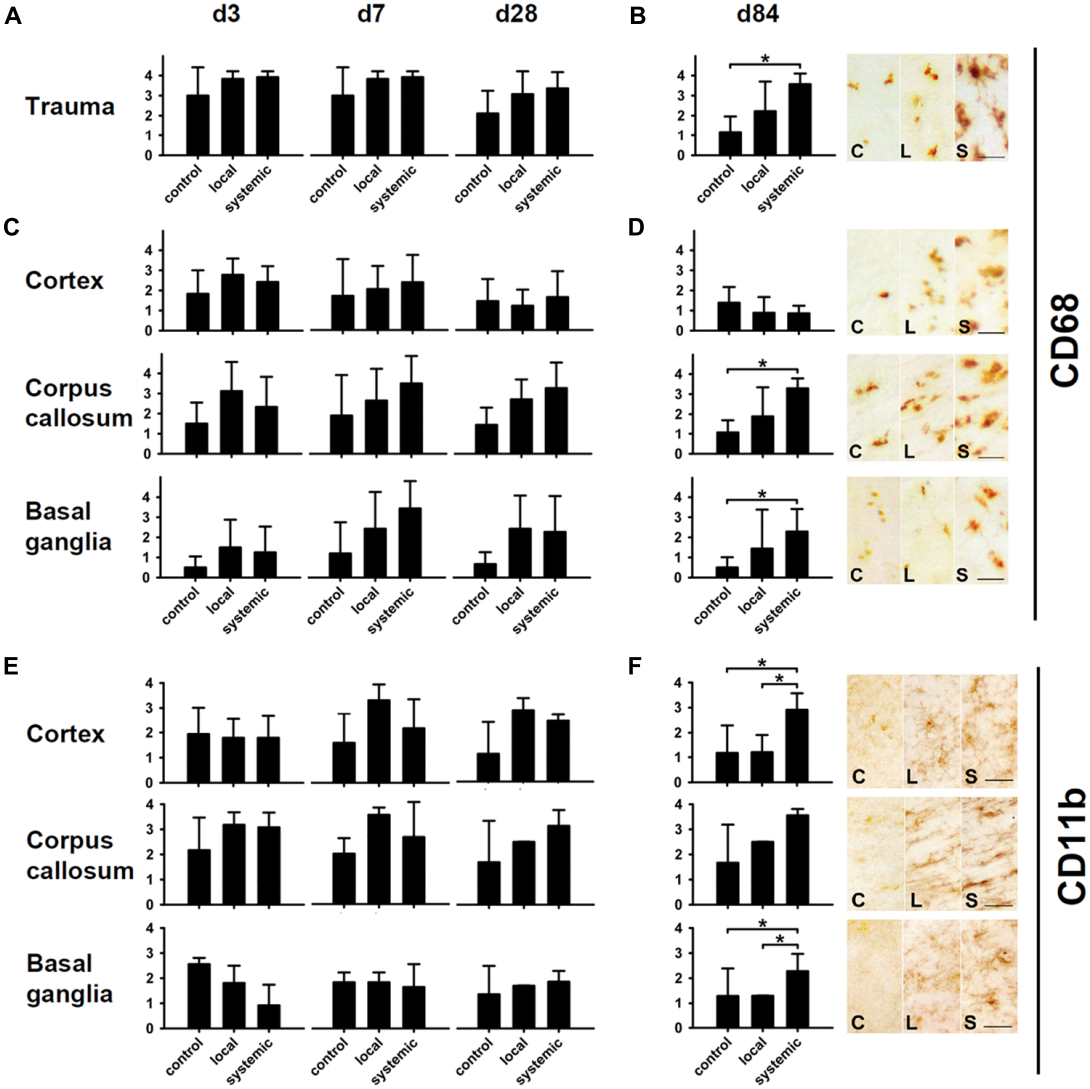
**Macrophage and microglia activity after hNPC transplantation.** Macrophage density as indicated by CD68 staining was increased at all investigated time points in animals receiving local or systemic hNPC transplantation **(A–D)**. Statistically significant differences were observed at 84 days after systemic cell transplantation in the area of the trauma, the corpus callosum and the basal ganglia **(B,D)**. A similar picture was observed for CD11b microglia. Apart from basal ganglia at early stages, highest mean numbers of these cells were observed at all investigated time points in both treatment groups **(E,F)**. Significantly enhanced numbers of microglia were detected 84 days after systemic cell transplantation in the perilesional cortex and the basal ganglia **(F)**. *p < 0.05.

**FIGURE 7:**
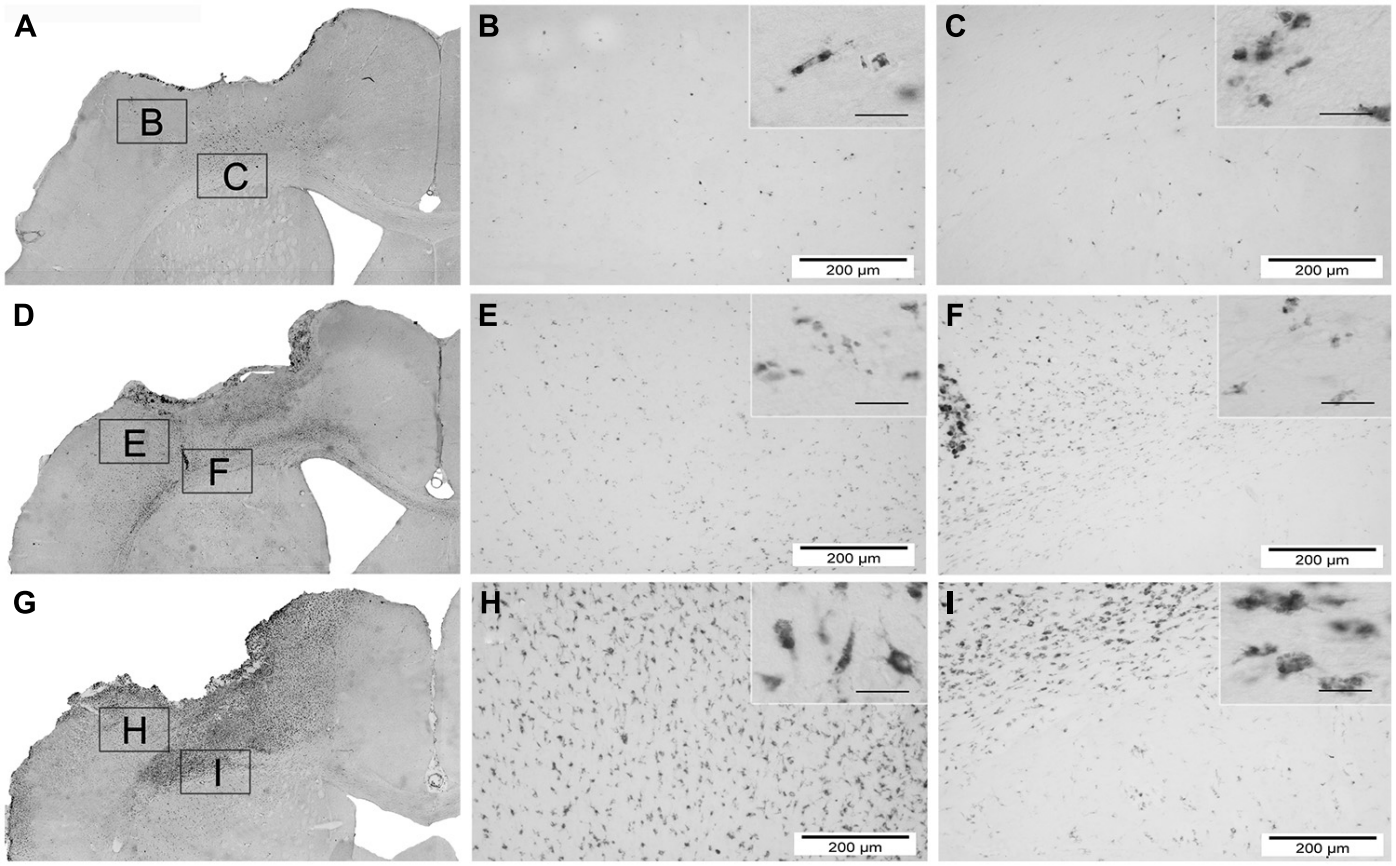
**Overview of macrophage activity (CD68) 84 days after hNPC transplantation.** photomicrographs depicting infiltration of CD68 positive cells of one animal of each group 84 days after brain injury and transplantation. Overview images are acquired by fusion of 9 single images of 2.5x magnification. Control group **(A–C)**, local transplantation **(D–F)** and systemic transplantation **(G–I)**. Insets of higher magnification (scale bars = 40 μm) on the right upper part of each image reveal details of the single cells. It is obvious that the systemically transplanted show the highest number of CD68 positive cells and the control the lowest.

CD68 (ED1) and CD11b (Ox42) antibodies recognizing microglia and macrophages were used to assess neuroimmunological reactions after therapeutic cell transplantation (**Figure 6**). Treated animals showed higher amounts of CD68-positive macrophages in corpus callosum, basal ganglia and trauma-affected cortical areas (**Figures 6A,C**). At d84, this difference was statistically significant for animals receiving systemic transplantation (*p* < 0.05 each, **Figures 6B,D**, for overview see also **Figure 7**). In contrast, no major differences were observed in the perilesional cortex with the number of CD68 cells slightly decreasing from d3 and/or d7 values toward d84 (**Figures 6C,D**).

**FIGURE 8:**
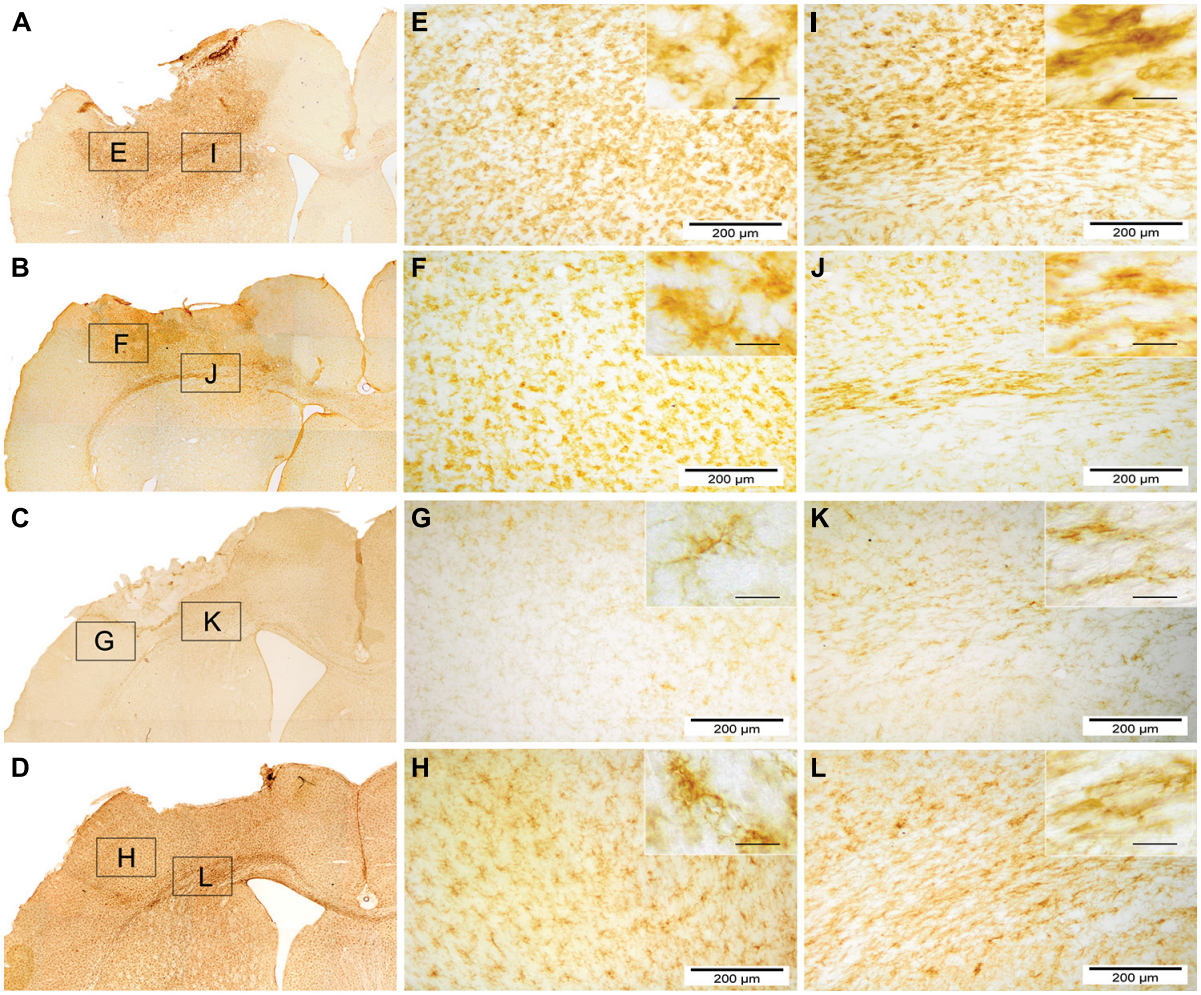
**Overview of microglial activity (CD11b) 7 and 84 days after local and systemic hNPC transplantation.** CD11b stained photomicrographs of locally and systemically transplanted animals 7 days and 84 days after transplantation. **(A–D)** display an overview of a locally transplanted animal 7 days **(A)** and 84 days **(C)** as well as a systemically transplanted animal 7 days **(B)** and 84 days **(D)** after transplantation. **(E–L)** depict the border zone beneath the impact area **(E–H)** and the corpus callosum with the adjacent cortex and putamen **(I–L)**. Insets show details of each subgroup at high power (scale bars = 40 μm).

Assessment of microglial reactivity (CD11b+) revealed inter-regional differences at d3 following TBI (**Figure 6E**). At d7, most pronounced microglial activation was observed after local hNPC transplantation in the cortex and corpus callosum (**Figure 6E**, for overview see also **Figure 8**). During later stages, this picture changed to higher CD11b expression in animals receiving systemic cell transplantation. In this group, differences were statistically significant in the perilesional cortex and basal ganglia (*p* < 0.05, **Figure 6F**) with highest density of CD11b cells being observed in all assessed regions. Unfortunately, analysis of the corpus callosum was confounded by a technical incident rendering the majority of samples from d84 after local transplantation (*n* = 7/10) unfeasible for analysis.

#### Impact of cell transplantation on post-traumatic astrogliosis

**FIGURE 9:**
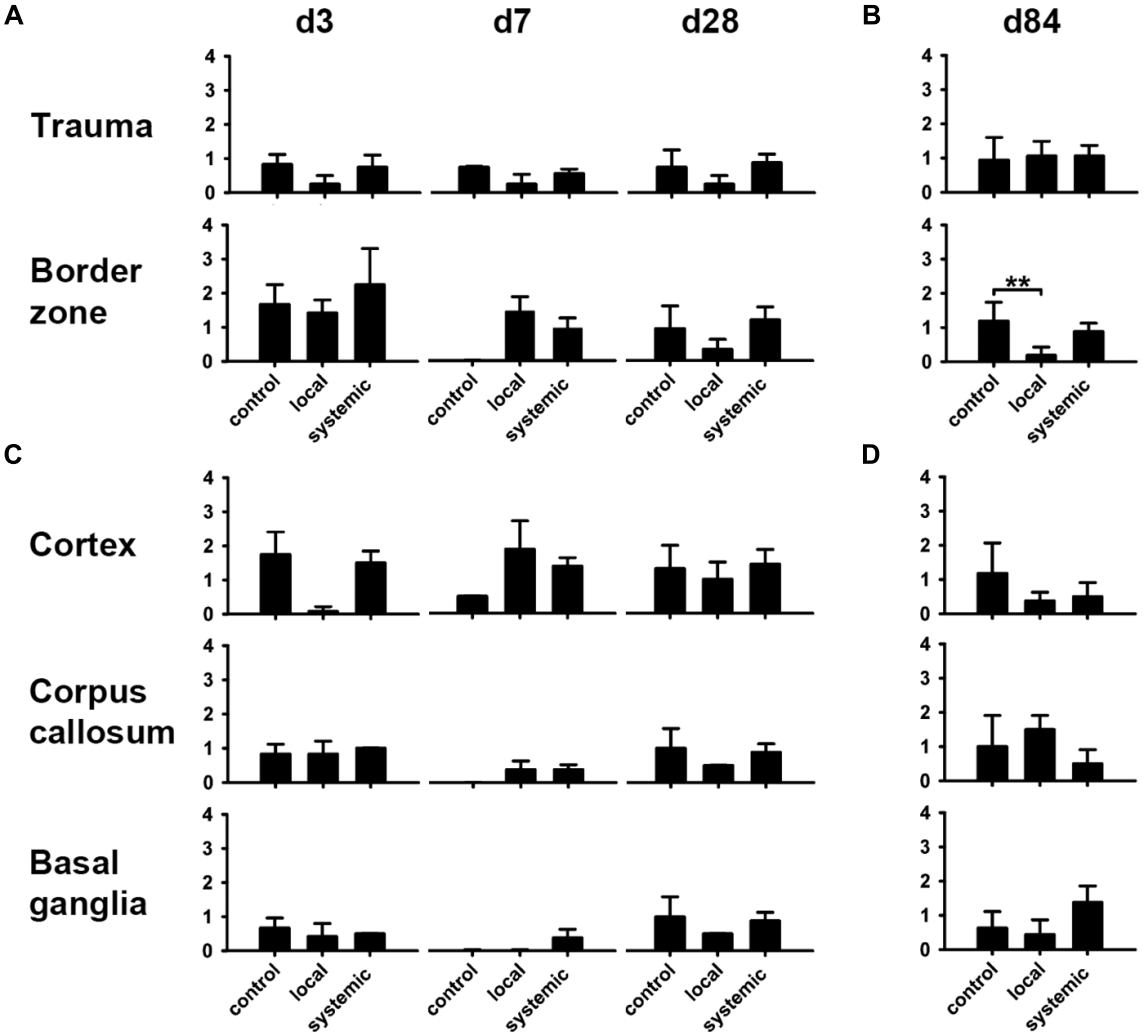
**Modulation of astrogliosis by hNPC treatment.** astroglial activation patterns showed a heterogenous phenotype. There was a tendency toward reduced astroglial activity in most investigated areas in animals receiving local hNPC administration, particularly at days 28 and 84 in trauma and border zones **(A,B)** as well as the perilesional cortex, corpus callosum and basal ganglia **(C,D)**. However, this difference was only statistically significant in the lesion border zone at day 84 **(B)**. **p < 0.01.

Analysis of reactive gliosis revealed a highly heterogenous pattern between different locations and groups. In general, astrogliosis was most pronounced in areas next to the lesion such as the perilesional cortex and the lesion border zone (**Figures 9A,C**), but surprisingly not cortical areas directly affected by TBI (**Figure 9A**). Local hNPC-transplantation significantly reduced astrogliosis in the border zone at day 84 (*p* < 0.01, **Figure 9B**). This result was not replicated in other investigated regions at day 84 (*p* > 0.05, **Figure 9D**), while astrogliosis was indifferent in the cortical areas affected by the trauma (*p* > 0.4, **Figure 9A**)

## DISCUSSION

This study was designed to evaluate the long-term impact of hNPC transplantation on the functional outcome after TBI, comparing intracerebral (stereotaxic) and intravenous transplantation paradigm. The cells were transplanted in the subacute stage 24 h after brain injury. This early time-point was chosen to take advantage of potential neuroprotective effects mediated by the graft, knowing that cell survival and viability would be likely more compromised in the subacute than in a chronic stage after TBI. Immunosuppression was started immediately thereafter mimicking a potential clinical scenario. Sustained functional improvement has not been detected after either transplantation approach although systemic NPC administration was associated with a transient functional improvement. No histological signs for a therapeutic modulation or cell replacement have been observed. However, some evidence was collected for ongoing inflammatory responses in the brain potentially leading to graft rejection and/or additional tissue damage despite the continuous immunosuppression by CsA.

### SAFETY ASPECTS OF NPC TRANSPLANTATION AND ASSESSMENT OF ADMINISTRATION ROUTES

Both treatment paradigms were safe without significantly enhanced mortality or reduced functional outcome among groups. However, individual performance was lowest during the early observation period after local transplantation. Although stereotaxic administration allows accurate placement of stem or progenitor cells in the target region and may, potentially, lead to higher concentrations of therapeutic cells and thereby enhanced cellular restoration, we did not found evidence for any of such processes in our study. However, the surgical intervention may have caused some transient but additional impairment without improved long-term outcome, advising caution regarding invasive cell administration procedures at least in the subacute stage following TBI. In turn, systemically administered hNPC may get stuck in pulmonary capillaries during the lung passage as shown for mesenchymal stem cells (MSC; Schrepfer et al., 2007). The MSC soma diameter (15–19 μm) is significantly larger than the one of murine pulmonary capillaries (5–8 μm; Schrepfer et al., 2007) and the soma volume of NPCs is comparable to MSC, exceeding that of circulating mononuclear leukocytes by an order of magnitude (Fischer et al., 2009). This confirms the assumption that cells can be filtered in the pulmonary capillary bed after systemic administration although a potentially related adverse effect on pulmonary function is left for further investigation. Other peripheral organs such as the spleen or the kidneys may also filter the systemically administered cells (Fischer et al., 2009). Interestingly, lower densities of NeuN-positive cells were observed in both, locally and systemically transplanted subjects at day 84. This may indicate a delayed immunological reaction against neuronal cell populations despite immunosuppression, which may even go beyond xeno-graft removal. This would also be in line with observed mid- and long-term indications of immunoreactivity after local and systemic cell transplantation, respectively. However, the lack of further data renders this assumption purely speculative and strongly suggests further investigation.

### EFFICACY OF NPC TRANSPLANTATION

Experimental studies using a variety cell types and transplantation paradigms have shown to improve the functional outcome following TBI within 2 weeks after cell transplantation (Harting et al., 2008; Maegele and Schaefer, 2008). Accordingly, we detected a clear functional improvement after systemic transplantation in the rotarod test with best performance observed during the medium-term observation period (2–6 weeks). However, this effect subsequently vanished, what was accompanied by signs of delayed induction of microglial activation and macrophage recruitment, both representing hallmarks of neuroinflammation following TBI (Schwab et al., 2002). No functional benefit by either transplantation paradigm was evident at 12 weeks, representing an overall neutral result regarding this clinically decisive outcome parameter.

### MECHANISMS OF CELL BASED FUNCTIONAL IMPROVEMENTS

Many groups were unable to detect any cells at later stages after local or systemic transplantation. Hence, functional recovery is unlikely to be explained by cellular restoration. Moreover, there is evidence that functional improvement is not necessarily related to cell engraftment (Lu et al., 2002; Harting et al., 2008) and particularly early functional improvements are more likely explained by neuroprotective or -modulative influences. For example, angiogenesis plays a crucial role during post-traumatic neuroprotection, in particular by restoring oxygen and nutrient supply and enabling immune cells to invade the lesion and to remove cell debris (Beck and Plate, 2009). Angiogenesis can already be detected within 3 days after ischemia and is induced by angiogenic factors such as VEGF and bFGF, which may even be secreted by cells outside the brain (Chen et al., 2005). Although not statistically significant, we observed a slight increase of RECA+ vessels in animals receiving hNPC transplantation. This was most prominent at d21, but not at d84 following systemic cell administration. Hence, observed transient functional improvements may be related to the release of angiogenic and potentially neurotrophic factors (Lu et al., 2003; Gao et al., 2006), but were not perpetuated because hNPC administration did not exert a significant long-term effect on cerebral microvessel density.

### NEUROIMMUNOLOGICAL ASPECTS OF LOCAL PROGENITOR CELL TREATMENT

**FIGURE 10:**
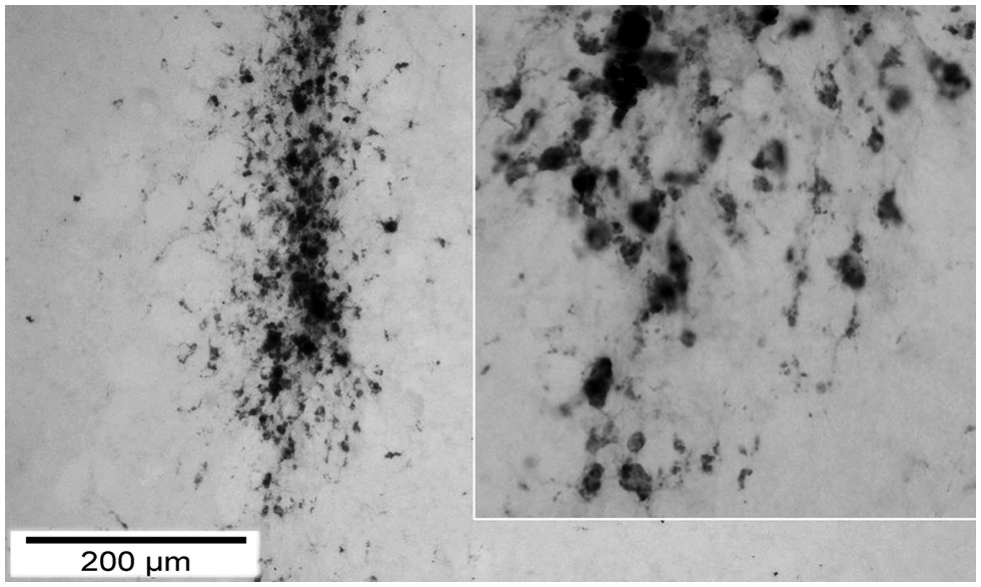
**Macrophage (CD 68) infiltration around the injection canal 7 days after TBI.** demonstration of the injection canal by CD68 staining 7 days after local transplantation. One can observe a massive local infiltration of CD68 positive cells. On the right the inset displays the tip of the canal in a higher magnification to show details of single cells.

We observed a significant increase of reactive microglia and macrophages particularly 12 weeks after trauma and transplantation. Although microglia are not primarily antigen presenting cells, they belong to the monocyte-macrophage system and are key players within the cerebral innate immune system (Mrass and Weninger, 2006). After brain injury, microglia become activated while macrophages are recruited to the brain parenchyma within 24 h. The least favorable functional performance was observed within the first 2 weeks after both TBI and local transplantation, potentially related to the additional trauma caused by local cell transplantation. Indeed, CD68+ and CD11b+ cells were transiently increased between d3 and d7 in most investigated regions, particularly in those regions situated in the vicinity of the lesion. Although the brain is considered an immune-privileged organ in its steady state, a stereotactically implanted cell graft is located in an immunologically active environment. Next to immune stimuli arising from the immediate cell death and tissue damage, an immunological activation can be triggered and fostered by BBB disruption, massive infiltration of antigen-presenting cells, an up-regulation of MHC-class II, altogether promoting recruitment and activation of T-cells and eventually graft rejection (Holmin and Mathiesen, 1999; Neumann, 2001; Galea et al., 2007). Consequently, it was shown by Shindo et al. (2006) that the survival rate of the graft highly depends on micro-environmental conditions with negative correlation to the severity of trauma and time to transplantation. Potentially, craft-borne cell debris may represent a strong stimulus to local microglia and recruited macrophages, also inducing removal of surviving cells within the graft at later stages. Indeed, we were not able to detect any transplanted cell but observed a strong infiltration of macrophages around the injection canal (**Figure 10**).

Widespread amelioration of astrogliosis was shown to correspond to improved functional outcome after CNS injury (Boltze et al., 2012). Local transplantation of hNCP induced a long-term decrease of astrogliosis in the lesion border zone, but not in other investigated areas. Thus, this effect may be too limited to translate into a robust therapeutic benefit, particularly in the context of above-mentioned neuroimmunological sequelae of local hNPC transplantation.

### LIMITATIONS OF THE APPLIED METHODS AND PROTOCOLS

This study comes with a number of limitations arising from methodological aspects, deserving a critical discussion. In particular, the failure to detect any surviving cells 84d after transplantation may be related to the applied xenotransplantation paradigm in combination with insufficient immunosuppression.

It has been shown independently from our experiments that NPCs can survive after xenotransplantation in the rodent environment under the same immunosuppressive regime and even without any immunosuppression (Schwarz et al., 2006), which is believed to be related to the immunological naivety of the cells as well as immunomodulatory capabilities (Gao et al., 2006; Mosher et al., 2012). However, the constellation of results obtained in our study questions this assumption. Ongoing graft rejection or even host tissue damage may be reflected by the overall increased numbers of CD68+ and CD11b+, which was statistical significant after systemic transplantation (**Figure 6**), as well as the reduced numbers of neuronal cells in transplanted animals (**Figure 5**) 84 days after transplantation.

The CsA mode of action is blocking of the nuclear factor of T cells (NFAT) via calcineurin inhibition. Since NFAT is a key regulator of pro-inflammatory cytokine expression (Kiani et al., 2000), CsA effectively mitigates inflammatory responses and selectively impairs T cell proliferation (Halloran et al., 1999). However, local (microglia) and peripheral (monocytes) phagocytotic cells are unaffected by CsA. It may hence be speculated that the innate immune system, particularly after BBB breakdown, is sufficient to eliminate the xenograft. Although stem cell populations can exert a therapeutic benefit beyond differentiation and functional integration (Oki et al., 2012), the rejection of the transplant may have accounted to the overall neutral study results. Further research should consider this possibility; especially since CsA application is a very common protocol for immunosuppression in experimental studies.

## CONCLUSION

Our results underscore the fundamental importance of long-term observation periods during the evaluation of experimental treatment strategies for TBI. Short observations periods may provoke an over- or underestimation of the therapeutic effect, which can at least partly be attributed to the extended time course of CNS lesion pathophysiology and reorganization. In our study, we would have overestimated the therapeutic effect of hNPC transplantation if the observation was limited to 4–6 weeks, which is common in experimental studies. Therefore, future studies should be long enough to reliably depict the pathophysiological steady state after CNS injury. This conclusion is supported by studies from other areas of experimental cell therapies for CNS disorders such as ischemic stroke (Balseanu et al., 2014). On the other hand, common practice in immunosuppressive treatments may require careful consideration during the design of such long-term experiments as non-autologous grafts may be subject to rejection despite implementation of the commonly applied CsA immunosuppression paradigm. Hence, we suggest not only a thorough evaluation of clinically relevant endpoints and experimental protocols, but observation periods being sufficient to reflect the final outcome of the disease model in order to detect late effects of any therapeutic intervention.

## AUTHOR CONTRIBUTIONS

Marco Skardelly conception of the work, analysis and interpretation of data and drafting the work, Khaled Gaber: acquisition and analysis of data and revising the paper, Swen Burdack: acquisition and analysis of data and revising the paper, Franziska Scheidt: acquisition and analysis of data and revising the paper, Martin U. Schuhmann: conception of the work and revising the paper, Heidegard Hilbig: analysis and interpretation of data and revising the paper, Jürgen Meixensberger: conception of the work and revising the paper, Johannes Boltze: discussion on conception of the work, data analysis and interpretation and drafting the work. All authors gave their final approval and agreed to all aspects of the work.

## Conflict of Interest Statement

The authors declare that the research was conducted in the absence of any commercial or financial relationships that could be construed as a potential conflict of interest.
